# Thermomechanical controls on magma supply and volcanic deformation: application to Aira caldera, Japan

**DOI:** 10.1038/srep32691

**Published:** 2016-09-13

**Authors:** James Hickey, Joachim Gottsmann, Haruhisa Nakamichi, Masato Iguchi

**Affiliations:** 1 School of Earth Sciences, University of Bristol, Bristol, BS8 1RJ, UK; 2 Sakurajima Volcano Research Center, Kyoto University, Kagoshima, 891-1419, Japan

## Abstract

Ground deformation often precedes volcanic eruptions, and results from complex interactions between source processes and the thermomechanical behaviour of surrounding rocks. Previous models aiming to constrain source processes were unable to include realistic mechanical and thermal rock properties, and the role of thermomechanical heterogeneity in magma accumulation was unclear. Here we show how spatio-temporal deformation and magma reservoir evolution are fundamentally controlled by three-dimensional thermomechanical heterogeneity. Using the example of continued inflation at Aira caldera, Japan, we demonstrate that magma is accumulating faster than it can be erupted, and the current uplift is approaching the level inferred prior to the violent 1914 Plinian eruption. Magma storage conditions coincide with estimates for the caldera-forming reservoir ~29,000 years ago, and the inferred magma supply rate indicates a ~130-year timeframe to amass enough magma to feed a future 1914-sized eruption. These new inferences are important for eruption forecasting and risk mitigation, and have significant implications for the interpretations of volcanic deformation worldwide.

The location and magnitude of crustal magma accumulation has important implications for volcanic hazards, eruption forecasting and risk mitigation^1^. It is possible to infer first-order estimates of these parameters using spatial patterns in geodetic data and generic models^2^,^3^. However, for more informed constraints, that are consistent across independent data sets, the inclusion of additional geophysical and geological observables are essential, necessitating the development of more sophisticated models. This is particularly pertinent when assessing if a volcano is escalating towards initial, renewed, or heightened eruptive activity. The latter may currently be the case at Aira caldera, where ongoing uplift signifies major concerns for volcanic hazard and risk assessment. Here, we show that by combining seismic tomography, topographical data, thermomechanical constraints and seismicity patterns, with spatial and temporal deformation observations, we can infer the location, volume, rate, timing, and mechanism of magma supply.

Aira caldera is located within Kagoshima Bay, at the southern end of Kyushu, in Japan (Fig. 1). Southern Kyushu has a rich volcanic history, with over 1000 km^3^ of andesite and dacite erupted in the past 1 M.a., and a series of calderas running through the island along a NNE-SSW trend^4^,^5^. Volcanism in this region is the result of fluid release from the subduction of the Phillipine slab^6^, and basaltic underplating supplying heat to melt the crust and generate felsic magmas^7^. The 17 km × 23 km wide Aira caldera was formed during a VEI (Volcanic Explosivity Index) 7 eruption ~29,000 years ago, which ejected ~98 km^3^ of pumice and produced a pyroclastic flow of ~300 cubic kilometres^4^,^8^. Petrological constraints indicate that the magmas feeding the caldera-forming eruption were stored at pressures of 0.3–0.5 GPa^9^ (~11–18 km depth), which is consistent with Sr-Nd measurements that indicate the middle-upper crust was subject to large-scale partial melting during magma formation^10^. Sakurajima, an active post-caldera andesitic stratovolcano, sits at the caldera’s southern rim (Fig. 1). It is Japan’s most active volcano with daily Vulcanian explosions emanating from the Showa Crater. Historical eruptive records are also punctuated with larger events, and in 1914 the ‘Taisho’ Plinian eruption (VEI 4) ejected 0.52 km^3^ of pumice and ash, followed by a 1.34 km^3^ lava flow^11^, killing 58 people. The syn-eruptive deflation caused coastal areas to be inundated with sea water, destroying rice fields, while the lava flows closed off the Seto straight, connecting the former island volcano to the mainland^12^.

Long-term deformation has been recorded at Aira caldera and Sakurajima volcano since 1892 with campaign levelling surveys (Fig. 1). Sporadic temporal sampling has been supplemented with data from a network of tide gauges and extrapolation of data from nearby sites to build up a detailed time-series^13^,^14^. Its most striking feature is the 1 m of subsidence that accompanied the 1914 paroxysm^12^, with the present-day uplift approaching the inferred level prior to that eruption^14^,^15^.

## The Role of Crustal Mechanics

The geodetic models used to date to explain the various periods of deformation at Aira caldera have all been based on the assumption of a simple, homogeneous, elastic, half-space^2^. Results indicate a deep deformation source 8–11 km beneath Aira caldera^2^,^14^,^16^,^17^,^18^,^19^, with a possible second source 3–6 km beneath Sakurajima^15^,^16^,^19^,^20^. The connection between the deep and shallow sources has been modelled as a NE-SW trending tensile fault^19^,^21^. However, mechanical heterogeneities in the crust are common in volcanic regions and cause variations in subsurface stress fields from a magmatic over-pressure, which translate to differences in modelled surface deformation when compared to a homogeneous medium^22^,^23^,^24^,^25^. Seismic velocity imaging indicates the crust at Aira caldera is particularly heterogeneous^6^,^26^,^27^,^28^,^29^. It is also likely that the crust is not entirely elastic: zones of low or zero seismicity (Fig. S1), as well as areas of high seismic attenuation^6^,^26^,^29^, could represent very hot or fractured material that does not respond in an elastic manner. Evidence for highly elevated temperature at depth is also seen in high surface heat fluxes^30^,^31^,^32^, a local geothermal gradient of 70 K/km^33^, and active submarine fumaroles within the Wakamiko depression^32^. Our model results indicate these three-dimensional mechanical and thermal effects are significant for the interpretation of volcanic geodetic timeseries.

We analyse Global Positioning System (GPS) data from 1996–2007 to infer the driving mechanism behind the uplift of Aira caldera (Methods). Contrary to previous studies^17^,^19^, we include both the horizontal and vertical deformation components, as well as an estimate of their associated error. The data delineate a radial outward bulge, centred within Aira caldera. To account for the known three-dimensional subsurface mechanical heterogeneity from the seismic velocity data (Fig. S2, Fig. S3, and Table S1), as well as surface topography, we use Finite Element (FE) analysis for our geodetic modelling (Fig. S4). In addition to the elastic three-dimensional crustal heterogeneity (termed 3D), we also compare one-dimensional and homogeneous model setups (termed 1D and HG, respectively) to demonstrate differences in results for simpler mechanical representations (Fig. S5). Furthermore, we assess the influence of including (TOPO), or excluding (HALF), topography and bathymetry, as the caldera depression and steep stratovolcano could induce surface strain localisation not accounted for with a flat half-space approach. This procedure produces a total of six different model classes. Within each model class, inversions were run using spherical, prolate, and oblate shaped deformation sources of varying size (Table S2) to obtain the optimal location and over-pressure to fit the surface displacement data (Methods)^34^.

## Strain Partitioning

To compare the six different model classes, the inversions results were evaluated against each other using the two-tailed students t-test (Table S3 and Methods). This is the first analysis of its kind to demonstrate and quantitatively assess the effect of heterogeneity and topography using an inverse approach. The results show statistically significant differences in source location (horizontal and vertical) for the different model classes (Fig. 2 and Table S3), and the best fits to the data were obtained with the 3D model class. This confirms that simple analytical models^2^ can not produce the same results as they are not able to capture the full complexity of processes (e.g., mechanical heterogeneity and topography) that influence surface deformation measurements. The overall effect of changing the subsurface structure (3D/1D/HG) is to alter the inferred location of a deformation source. This is due to the way strain is partitioned between relatively stiffer and softer regions of the crust. Subsequently, the variation in strain transfer between the source and surface manifests itself in different ground displacement patterns. This effect is particularly apparent when the softer regions are adjacent to irregular sections of the expanding deformation source^23^.

The way that subsurface stress or strain is partitioned is also dependent on the interactions between source shape and surface topology. When comparing the TOPO and HALF models, the most significant changes in source location and model fit are seen in the prolate and spherical shaped sources (Table S3). This is due to their radial strain, which partitions at the surface in a complex manner to alter the inferred horizontal source location. The oblate source locations are conversely least affected by the influence of topography, as they produce a higher degree of vertical strain. Generally, better fits to the data were achieved with the HALF model class when compared against the TOPO model class. This is likely due to non-unique model solutions and non-linear interactions between surface strain, subsurface strain and surface deformation, but we consider topography a prerequisite in our models as it improves the consistency with real-world observables.

Our results have significant implications for the interpretation of volcanic geodetic data worldwide. When including subsurface heterogeneity the most significant changes in model fit are observed for spherical shaped sources (Table S3), and spherical sources are also strongly affected by topography. Furthermore, better fits to the data are obtained using the HALF model class when compared to TOPO. Consequently, employing an oversimplified model with a popular spherical source shape (e.g., the ‘Mogi’ model)^2^ is likely to result in erroneous source locations due to the neglect of topography and realistic subsurface structure, despite goodness-of-fit criteria unjustifiably favouring such a model (i.e., the fit to the data is acceptable, but the source location and model are wrong). The amount of error will be proportional to the degree of subsurface heterogeneity and the irregularity of the surface topography.

The results of the 3D TOPO model inversions were used as a starting point to produce a best-fit model for the 1996–2007 inflation period (Methods, Figs S6 and S7), as this model class incorporates the mechanical conditions most consistent with other geophysical data. The final best-fit source is oblate in shape and located in the north-east of Aira caldera at a depth of 13.1 km (Fig. 3 and Table 1). Its over-pressure of 2.7 MPa is comfortably within the *in-situ* rock tensile strength estimates often used as maximum values for magma reservoir pressurisation (0.5–9 MPa)^35^,^36^, and translates into a volume increase of ~1.2 × 10^8^ m^3^. This volume change is consistent with the results of analytical models for the same deformation period^17^,^19^, but the source location has changed substantially (Fig. 3 and Table 1). Our best-fit source is deeper, and located in the north-eastern quadrant of the caldera, beneath a low-velocity zone (Fig. S3) and numerous volcano-tectonic (VT) hypocentres (Fig. S1), as well as beneath the Wakamiko depression and its associated fumaroles.

This proposed source location provides a more consistent interpretation of the local volcanological and geophysical observations. The deformation source likely represents a magma reservoir, which supplies heat and fluids to the shallow subsurface system. These processes simultaneously reduce the seismic velocity above the reservoir, feed the Wakamiko fumaroles, and contribute to the local volcano-seismicity. A source closer to Wakamiko is also consistent with He isotope measurements, which indicate that Sakurajima and the fumaroles have the same magmatic source^32^. Furthermore, the depth of the reservoir fits with petrological observations of mid to upper crustal magma storage^9^,^10^. The difference in source location is primarily driven by the inclusion of a three-dimensional mechanically heterogeneous subsurface and resultant changes in strain partitioning. This highlights the fundamental control of crustal mechanics on the location of magma supply. The inferred reservoir is quite large, and the modelled oblate shape might be representative of a collection of melt lenses or a distributed network of sills and dykes^37^.

When inspecting the best-fit modelled deformation vectors there are two stand-out mismatches: the horizontal displacements modelled at sites FUTG and KURG are very dissimilar to those observed in the GPS results (Fig. 3). A possible explanation for this is that a magma pathway or dyke, aligned NE-SW, exists between the best-fit source within Aira caldera, and a smaller and shallower short-term magma storage region beneath Sakurajima. Magma flowing in this pathway would cause a pressurisation to create large horizontal displacements in the NW-SE direction, and more like the deformation observed at FUTG and KURG. A similar mechanism has previously been proposed^21^ due to the NE-SW alignment of faults. Our work is in agreement with this interpretation where magma exploits structural weaknesses in the crust to travel from a storage reservoir within Aira caldera to beneath Sakurajima, prior to eruption. This highlights the potential for substantial lateral transport of magma beneath calderas before eruption.

Anomalous local GPS site-effects at MAKI and KIHO may also contribute to reducing the overall goodness-of-fit. Their measured GPS deformation vectors do not correlate well with the overall inflation pattern (Fig. 3), due to their very close proximity to regional and caldera faults^4^. The FE models employed do not account for faulting related processes, which can act as strain barriers^38^ and may reactivate due to magmatic accumulation^39^, and could provide an explanation for the discrepancy between modelled and observed deformation vectors.

## Thermomechanics and Magma Supply

Southern Kyushu has a significant thermal signature with high heat flow values^30^,^31^,^32^ that preclude elastic crustal conditions, a consequence of millions of years of magmatic accumulation and eruption^40^,^41^. To account for the resulting elevated geothermal gradient, inelastic rock behaviour^42^ and thermomechanical strain relaxation, as well as the temporal inflation pattern, we apply the best-fit source parameters from the inversions in a suite of forward FE models that incorporate a temperature-dependent viscoelastic (TDVE) rheology (Methods). To fit the same spatial deformation pattern as the elastic models the source overpressure can be reduced by a third, to 1.8 MPa. The application of a constant pressure-time function strongly demonstrates the influence of the viscoelastic deformation; following an instantaneous elastic inflation the viscous creep continues to generate uplift whilst the pressure function is flat (Fig. S8). However, this does not fit the temporal deformation pattern. Instead, we use a simple ramped pressure-time history to provide a sufficient fit^23^,^25^,^43^ (Fig. 4). The amplitude of the modelled deformation at the GEONET^44^ site is slightly over-estimated with the 1.8 MPa pressure, but this requirement is justified by the good fit at other sites. By scaling the deformation at the GEONET site we show that the modelled temporal inflation rate matches the observed GPS displacements (Fig. 4). This includes some continued inflation whilst the pressure-time function is flat (period 2001–2003, Fig. 4b), a result of the viscoelastic rheology and similar in effect but smaller in amplitude to the observed creep when applying a constant pressure-time boundary load (Fig. S8). The amplitude of this creep effect is smaller when applying a ramped pressure load because the viscous deformation during the period of increasing pressure is constantly ‘overwritten’ by the continued elastic deformation, so that when the pressure function is flat there is only a small proportion of viscous creep remaining to continue the inflation. This is the result of the Boltzmann superposition principle. Our models do not capture the full amplitude of minor inflation during the 2001–2003 period, and this might be improved by a non-linear viscoelastic rheological representation that produces a larger proportion of creep, or another physical process leading to reservoir pressurisation that our models do not capture (e.g., bubble growth). An elastic model utilising the same ramped pressure-time function would have a worse fit to the data as it would be unable to reproduce any inflation when the pressure function is flat.

**Figure 1:**
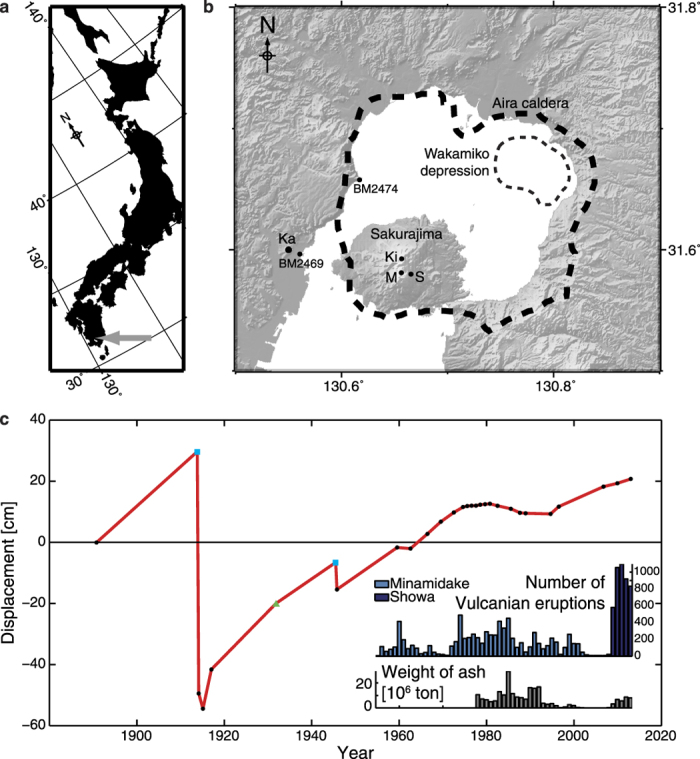
The location and deformation of Aira caldera. (**a**) Aira caldera is situated at the southern end of Kyushu (Japan), indicated by the grey arrow. (**b**) The geographical relationship between Sakurajima volcano, Aira caldera and the Wakamiko depression. *Ka* - Kagoshima city; *Ki* - Kitadake (summit); *M* - Minamidake, *S* - Showa crater (recent eruptive vents); *BM* - levelling benchmarks. (**c**) Long-term caldera deformation; the movement of levelling benchmark 2474 relative to 2469 (black dots). The blue squares and green triangle indicate where data has been inferred from tide gauge data or extrapolation from other levelling benchmarks respectively (see refs 13 and 14 for more details). Insets show the eruptive activity from the Showa crater and Minamidake vents on the same time axes. Maps created using GMT 4^59^.

**Figure 2:**
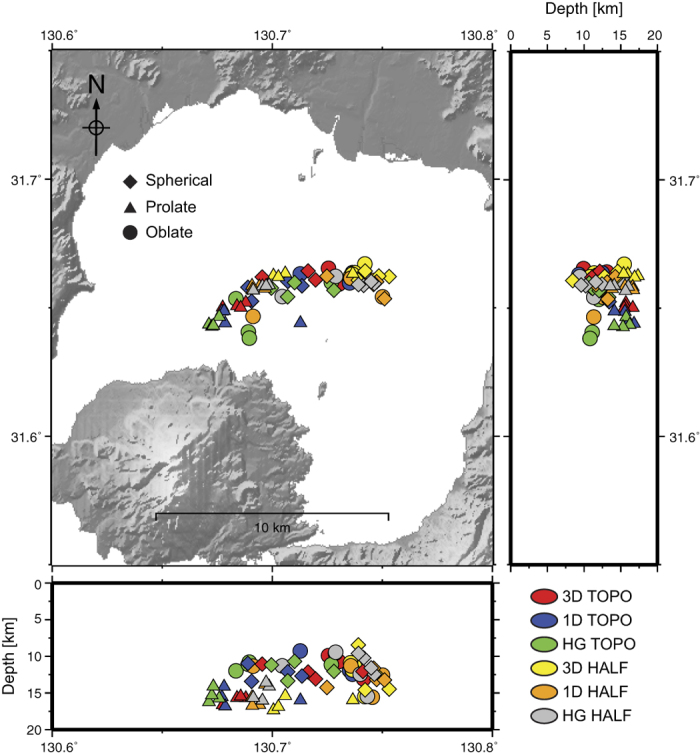
Inversion source locations. The coloured shapes indicate the converged source locations from the final iteration of each inversion, for the range of source sizes considered in the statistical tests (Methods). All three tested source shapes are displayed. The different colours represent the six different model classes. Map created using GMT 4^59^.

The updated modelled volume change is 1.38 × 10^8^ m^3^, due to the additional viscous volume expansion. The total mass erupted over the same period is 7.85 × 10^9^ kg (Fig. 1). Assuming a dense rock equivalent (DRE) magma density of 2500 kg/m^3 ^45, this equates to a volume of 3.14 × 10^6^ m^3^. Hence, we can estimate that the total amount of magma supplied to the system (stored plus erupted) is 1.41 × 10^8^ m^3^. Over the 10-year period modelled, this is equivalent to an average long-term magma supply rate of 0.014 km^3^/yr. When examining the two deformation pulses individually, these equate to short-term supply rates of ~0.02 km^3^/yr. A simple Mogi^2^ model would be unable to resolve these differences as it can not distinguish pulses in deformation or magma supply, and does not have any time-dependency. Both magma influx estimates are sufficient to supply and sustain a large magma reservoir for eruption in this region of elevated crustal temperatures, given minimum rates of only 0.01 km^3^/yr are required in cold conditions^46^.

With the observed and modelled temporal deformation patterns, rough constraints can be placed on the timing of magma supply. The results shown suggest that there were two discrete pulses, one from early-1997 to early-2000, and another from mid-2003 to mid-2006 (Fig. 4). A strong correlation between the deformation and local volcano-seismicity rates also supports this conclusion (Fig. S9). The pulses of magma supply are consistent with the view that large igneous bodies must grow through incremental accretion of multiple sill intrusions^47^,^48^, and the modelled oblate shape of the inferred magma reservoir corroborates this growth mechanism^49^. Here, our models have inferred the timing and mechanism of magma supply – two parameters unobtainable with simple analytical methods (e.g., the Mogi^2^ model).

Furthermore, the depth of the reservoir (~13 km) is significant for magma supply. At this level beneath the caldera, the temperature of the magma (~1273 K) is very similar to that of the surrounding host-rock (~1200 K, Fig. 4). The small temperature contrast maintains the system at near thermal equilibrium, so minimal heat is lost and may explain the continued growth and sustainment of the reservoir. In contrast, if the reservoir was located at 10 km depth, the surrounding temperature would be colder (~980 K), and the magma would cool down faster.

## Reservoir Priming

The magmatic evolution of the Aira caldera system appears to have been dictated by magma supply through numerous sill intrusions, and maintained at eruptible conditions in a mid-crustal hot zone by the high magma flux and low temperature contrast with the host-rock. Magma is currently being supplied faster than it is erupted, which explains the continued long-term inflation beyond the period studied in detail here (Fig. 1). The volume of eruptible magma is a key parameter when considering an increase in eruptive/explosive activity and the possibility for a future eruption similar to, or larger than, the 1914 VEI 4 event.

The 1914 eruption produced a total volume of 1.86 km^3^ (0.52 km^3^ of tephra and 1.34 km^3^ of lava)^11^. Using a tephra density of 1500 kg/m^3 ^50, an average lava density of 2200 kg/m^3 ^12 and a DRE density of 2500 kg/m^3 ^45, this is equal to a total DRE volume of 1.49 km^3^. Through the period from 1978 (when the record began) to 2013, ~223 × 10^9^ kg was erupted, or 8.9 × 10^7^ m^3^ DRE, which equates to an eruption rate of ~2.55 × 10^6^ m^3^/yr. Assuming the long-term supply rate inferred from this study (0.014 km^3^/yr) is roughly constant, we can subtract the eruption rate to estimate a magma storage rate of 1.15 × 10^7^ m^3^/yr. This storage rate implies a timeframe of ~130 years to re-accumulate enough magma for another 1914-sized eruption. Similarly, the previous large-scale ‘An’ei’ eruption occurred between 1779 and 1782. Applying the same rates to the period immediately following this eruption would hindcast a 1914-sized eruption in ~1912; a remarkable result given the rough approximations taken into account.

Any alterations to the inferred magma supply or eruption rates would change the post-1914 estimate. For example, since 2006 there has been a marked increase in the number of eruptions, but the inflation rate has remained roughly constant (Fig. 1). This could represent an increase in the magma supply rate, with a subsequent decrease in the implied time to accumulate enough magma for a 1914-sized eruption. Evidently, a thorough understanding of the rate and volume of magma supply and accumulation, and their thermomechanical controls, is essential for continued monitoring and eruption forecasting at Sakurajima volcano, and volcanoes worldwide.

## Methods

### GPS Observations

**Figure 3:**
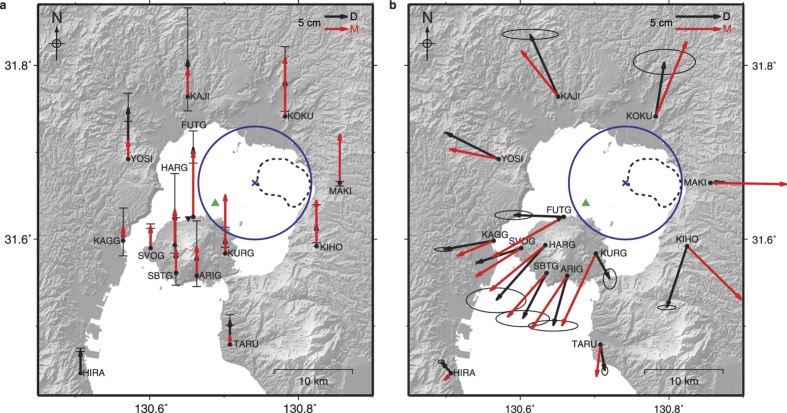
Best-fit model deformation vectors and source location. Black arrows indicate GPS measurements (1996–2007) with 95% confidence limits, while red arrows indicate the best-fit model surface displacements, for vertical (**a**) and horizontal (**b**) components. The blue circle shows the surface projection of the best-fit source from this study, with the cross indicating its centre (Table 1). For comparison, the green triangle is the analytical model source centre location for the same deformation period^17^. GPS station names are shown. The inverted black triangle indicates the location of a GEONET continuous GPS station^44^ and the dashed line represents the outline of the Wakamiko depression. Maps created using GMT 4^59^.

**Figure 4:**
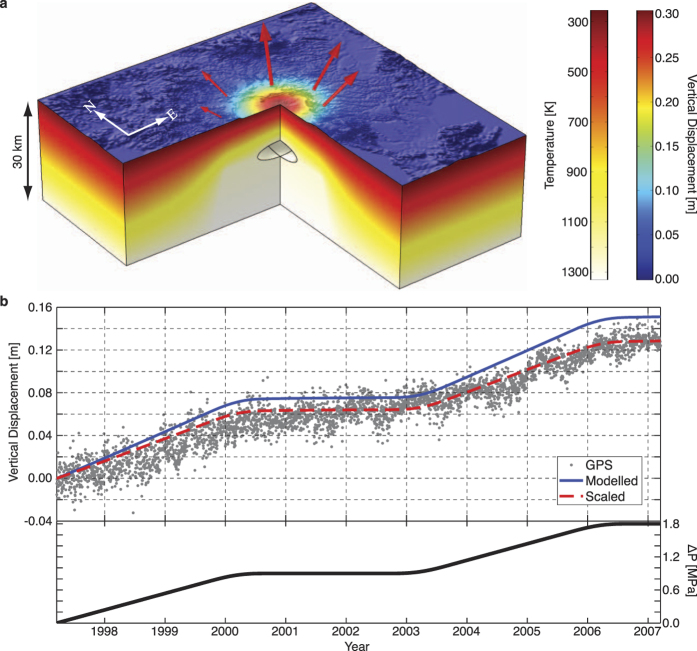
Thermomechanical modelling and temporal deformation. (**a**) Cross-section of TDVE model setup with best-fit source parameters. The coloured surface shows the modelled vertical deformation, while the red arrows show the modelled three-dimensional displacements at five GPS sites. The depth and source dependent temperature distribution is also shown. (**b**) (Top) Vertical GPS time series recorded at the GEONET site and modelled temporal inflation patterns. The blue line shows the model prediction using a simple ramp pressure-time function. By scaling this result to 85% (red line) it is clearer that the modelled temporal displacement rate matches the GPS observations. (Bottom) Pressure-time function used in the TDVE models. The ramp is curved slightly to ease the numerical computation within the FE model.

We examine the GPS derived surface displacements from the 1996–2007 period. A campaign GPS network exists over much of Aira caldera and Sakurajima volcano, and is maintained by the Sakurajima Volcano Research Centre^17^,^19^. Horizontal results for 1996–2007 have been presented before^17^ and allow for comparison, but here for the first time we also incorporate the vertical data and an estimate of their associated 95% confidence limits (Fig. 3). The spatial deformation pattern shows a radial outward bulge, centred within Aira caldera. Continuous GPS results have also been examined for the 1996–2007 period and show two discrete pulses in the deformation, but these are only horizontal baseline length-change time-series between two stations across the volcano (e.g., SVOG and KURG)^17^. Instead, to assess the temporal deformation pattern we use the continuous record of a GEONET GPS station^44^ (Fig. 3, inverted triangle west of FUTG), which provides a vertical time-series that can be used for the temporal deformation of the Aira caldera region.

### Model Setup

#### Geometry and mesh

The Finite Element (FE) software COMSOL Multiphysics (v4.4) is used to construct and compute the models developed in this study. A full 3D model geometry is used (Fig. S4). This allows us to incorporate the topography and bathymetry of the Kagoshima Bay region, as well as the seismic tomography data, which varies in three directions. To represent the deformation source we use a spherical, prolate or oblate-shaped cavity. An Infinite Element Domain (IED) is used to surround the model geometry which prevents boundary effects at the model limits affecting the results of the interior^24^. Tetrahedral-shaped elements are used for the majority of the model domain, with ‘swept’ prism-shaped mesh elements being used in the IED. The final model geometry is 88 km × 88 km × 30 km in size, made up of ~210,000 mesh elements, with higher mesh density around and above the source (Fig. S4). A modelled volume change was calculated within COMSOL by integrating over the three-dimensional expansion of the deformation source.

#### Elastic material properties

3D seismic tomography data^27^ is used to infer the mechanical properties of the subsurface (Fig. S3). V_P_ and V_S_ seismic velocities are converted to a Poisson’s Ratio, *ν*, density, *ρ*, and dynamic Young’s Modulus, *E*_*D*_ with the following equations^51^:


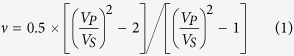



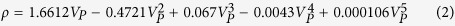



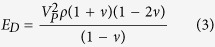


The static Young’s Modulus, *E*, is more appropriate in our case as the processes affecting volcanic deformation are much slower than seismic wave propagation^52^. *E*_*D*_ is 2–13 times larger than *E*^53^,^54^, so we use a conservative scaling factor of 2 to convert from *E*_*D*_ to *E*. The resulting values are appropriate for volcanic areas^52^ (e.g., Fig. S2). Other model parameters are as in Table S1. To test the effects of including a 3D subsurface material heterogeneity we used the same data to produce a vertically variable (1D) material profile, taking mean values at regular 1 km depth slices (Fig. S5). We also took overall mean values to calculate material properties representing a homogeneous (HG) medium. The three different subsurface material representations were used in the elastic inversion models. The tomography data we use is derived from a regional study^27^. Future studies might benefit from including the data of smaller-scale, higher-resolution local studies^6^,^26^,^28^,^29^ in combination with the regional data to allow for a more detailed parameterisation of the near-volcano region.

#### Elastic inversion models

Six different model classes were used. These employed a geometry either with or without topography and bathymetry (TOPO and HALF, respectively), in addition to one of the three different subsurface material representations (3D, 1D, or HG), e.g., 3D TOPO or 1D HALF. The boundary conditions were the same across all six, and were adapted to a 3D model geometry from benchmarked model configurations^24^; i.e., a fixed bottom surface, a free top surface, rollers on lateral surfaces and an IED surrounding the model geometry. The deformation source was given a boundary load (overpressure) normal to the source surfaces (Fig. S4). Improving upon a similar principle in an earlier application^34^, the inversion procedure works by minimising an objective function while searching for the optimum deformation source parameters (longitude, latitude, depth, overpressure) to fit the error-weighted GPS deformation data. Hence, the overpressure is not calculated, but the optimal overpressure-source location combination is determined. Each source parameter is given a defined range, and the algorithm varies them within this range, ‘walking’ to the best solution and automatically re-building the mesh on every iteration. The objective function, *J*, is expressed as:


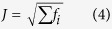


where,


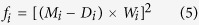



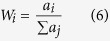



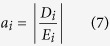


and *M*, *D*, *E*, and *W*, are the model, data, error and weight respectively, for the index, *i*, relating to the individual GPS vectors at each station. Hence, *J* is the square-root of the weighted residual sum of squares. The specific inversion search algorithm is called BOBYQA (Bound Optimisation BY Quadratic Approximation), a derivative-free numerical optimisation^55^.

#### Modelling strategy

The elastic inversion models were used to test the effects of topography and subsurface structure. For each inversion, the source location from an analytical model result of the same period of deformation was employed as an initial starting location^17^, with the exception of the prolate sources which had a deeper starting location to accommodate their vertically elongate shape, but the same horizontal coordinates. Holding the source shape constant, the same five inversions were run in each of the six different model classes, with the size of the source varying between the model runs (30 in total). The same procedure was used for all three source shapes. Hence, for each model class the same fifteen inversions were carried out to enable a thorough comparison between them (90 in total). The spherical sources had a radius, *R*, of 1–5 km. The oblate and spherical sources had equivalent volumes to the spherical sources, with their axes lengths determined by a fixed ratio between the vertical, *a*, and horizontal, *b*, components; 1:3 and 3:1, respectively (Table S2). Dipping sources were not considered due to the rough axisymmetry of the deformation pattern. To establish a best-fit model for the spatial deformation pattern, the results of the 3D TOPO inversions were further examined. The source parameters from this best-fit model were used in the temperature-dependent viscoelastic models to examine the temporal deformation pattern whilst better representing the mechanics of the crust beneath Aira caldera.

#### Temperature-dependent viscoelastic forward models

We adapt a benchmarked viscoelastic model^24^ to include temperature-dependent viscoelastic (TDVE) effects in a suite of two-step, 3D forward models (Fig. S4). A steady-state temperature distribution is solved first, using a surface temperature of 283 K, a magmatic temperature of 1273 K^7^, and a heterogeneous geothermal gradient. A value of 70 K/km is used beneath the caldera^30^,^33^, down to a level commensurate with the best-fit source. After this, the temperature here is increased at a rate of ~4 K/km, in order to match the temperature at the base of the crust (model domain) from the 34 K/km geothermal gradient of the larger region (applied at the external model boundaries)^33^. This fits with interpretation of basaltic underplating in the southern Kyushu region^7^, with a temperature at the bottom of the crust of ~1300 K. The temperature variable, *T*, is used to define the viscosity, *η*, with an Arrhenius formulation, where:


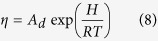


and *A*_*d*_ is the Dorn Parameter, 10^9^ Pa s^56^,^57^, *H* is the activation energy, 135 kJ/mol^58^, and *R* is the universal gas constant. This viscosity is used in the second step to calculate the deformation of the crust using a linear viscoelastic material model (Standard Linear Solid representation), instead of an elastic assumption. The solid mechanics boundary conditions are the same as those used in the elastic inversion models (Fig. S4) but the model now has an additional time-dependent element.

### Inversion result comparisons

The six different inversion model classes were evaluated against each other using the two-tailed students t-test. We use p-values less than 0.10 to indicate a statistically significant difference at the 90% level (Table S3). Results from the smallest volume sources (*V* = 4.19 km^3^) were removed from this analysis as their small size precluded a fit to the data with realistic source parameters (pressures too high/depths too shallow). Overpressure results are not compared as the range in source size and obvious trade-off between source size and overpressure prevents meaningful comparisons being made. It is noted, however, that the 3D model class required the smallest overpressures, and the HG model class required the largest. Using the misfit objective function, *J*, as an indicator of the overall effect of changing an aspect of the model setup, it is clear that the biggest effects are seen in the spherical sources when altering the subsurface heterogeneity (Table S3).

### Constraining the best-fit model

Oblate-shaped sources provided the best fits to the data with the smallest residual values, while prolate sources were the worst (Fig. S6). This is due to variations in the relative amounts of radial and vertical strain the different shaped sources produce, and the interactions these have with the three-dimensional mechanical heterogeneity. To constrain the individual best-fit model, the results from the optimum 3D TOPO inversion were further investigated, as this model setup represents the conditions closest to reality, i.e., we consider topography and subsurface heterogeneity a prerequisite for our models when searching for a best-fit source. The best model inversion result from this setup was the oblate-shaped source with *V* = 523.6 km^3^ (Table S2). Its source geometry was used as an input for a FE Monte Carlo simulation in which the *X*, *Y*, *Z*, and Δ*P* parameters were sampled randomly, and the mesh was automatically rebuilt on each iteration. The Monte Carlo simulation did not find a better combination of source parameters, so the result from the initial inversion was then used as an input for a subsequent inversion with a smaller, nested, parameter constraint grid (Fig. S7). This ensured the final model parameters were as robust as possible^34^. In the current case of 3D TOPO inversions with oblate-shaped sources, lower p-values in the t-tests show that heterogeneity was dominant over topography in altering the source location (Table S3: rows 19–21 *c.f.* row 25). Error estimates on the source parameters were evaluated by running Monte Carlo simulations with the upper and lower GPS uncertainties instead of the optimum values. As we did not test the infinite range in source shape and size, other models with equally good fits to the data may also exist.

## Additional Information

**How to cite this article**: Hickey, J. *et al*. Thermomechanical controls on magma supply and volcanic deformation: application to Aira caldera, Japan. *Sci. Rep.* **6**, 32691; doi: 10.1038/srep32691 (2016).

## Supplementary Material

Supplementary Information

## Figures and Tables

**Table 1:** Best-fit deformation source parameters for the 1996–2007 period from this study and a previous analytical modelling approach^17^.

	This Study	*Iguchi et al*.^17^
Longitude [°]	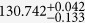	130.688
Latitude [°]	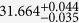	31.641
Depth to centre [km]	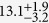	11
Overpressure [MPa]	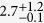	—
Volume change [m^3^]	1.2 × 10^8^	9 × 10^7^
Shape	Oblate	Spherical
Vertical semi-axis [km]	2.4	—
Horizontal semi-axes [km]	7.2	—
