# Supplementary material for: Thermomechanical controls on magma supply and volcanic deformation: application to Aira caldera, Japan
Source: Sci Rep. 2016 Sep 13;6:32691. doi: 10.1038/srep32691 (PMC5020646; doi:10.1038/srep32691)
Supplement: Supplementary Information [file srep32691-s1.pdf]

SUPPLEMENTARY MATERIAL FOR:

Thermomechanical controls on magma supply and volcanic deformation: application to Aira caldera, Japan

James Hickey[1*], Joachim Gottsmann[1], Haruhisa Nakamichi[2] & Masato Iguchi[2]

[1] School of Earth Sciences, University of Bristol, Bristol, BS8 1RJ, UK

[2] Sakurajima Volcano Research Center, Kyoto University, Kagoshima, 891-1419, Japan

[*] Now at: Camborne School of Mines, University of Exeter, Penryn Campus, Cornwall, TR10 9FE, UK.

Email correspondence to: j.hickey@exeter.ac.uk

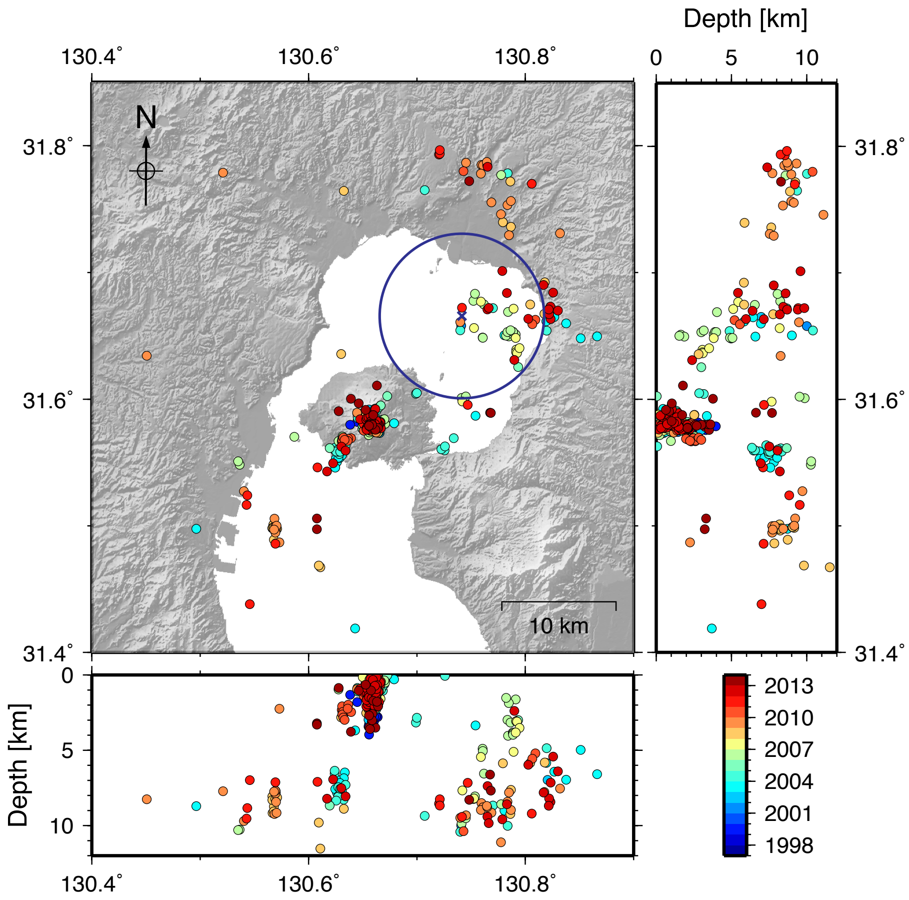

Supplementary Figure 1. Spatio-temporal distribution of volcano-tectonic (VT) seismicity. Aseismic zones are apparent in the north-west of the caldera as well as beneath Sakurajima's central conduit (> 5 km). Events are colour-coordinated by time. The events south-west of Sakurajima's summit (~130.62° E) have also been hypothesised to have a tectonic rather than volcano-tectonic origin[21]. The surface projection of the best-fit oblate source from this study is shown with the blue circle. Map created using GMT 4[59].

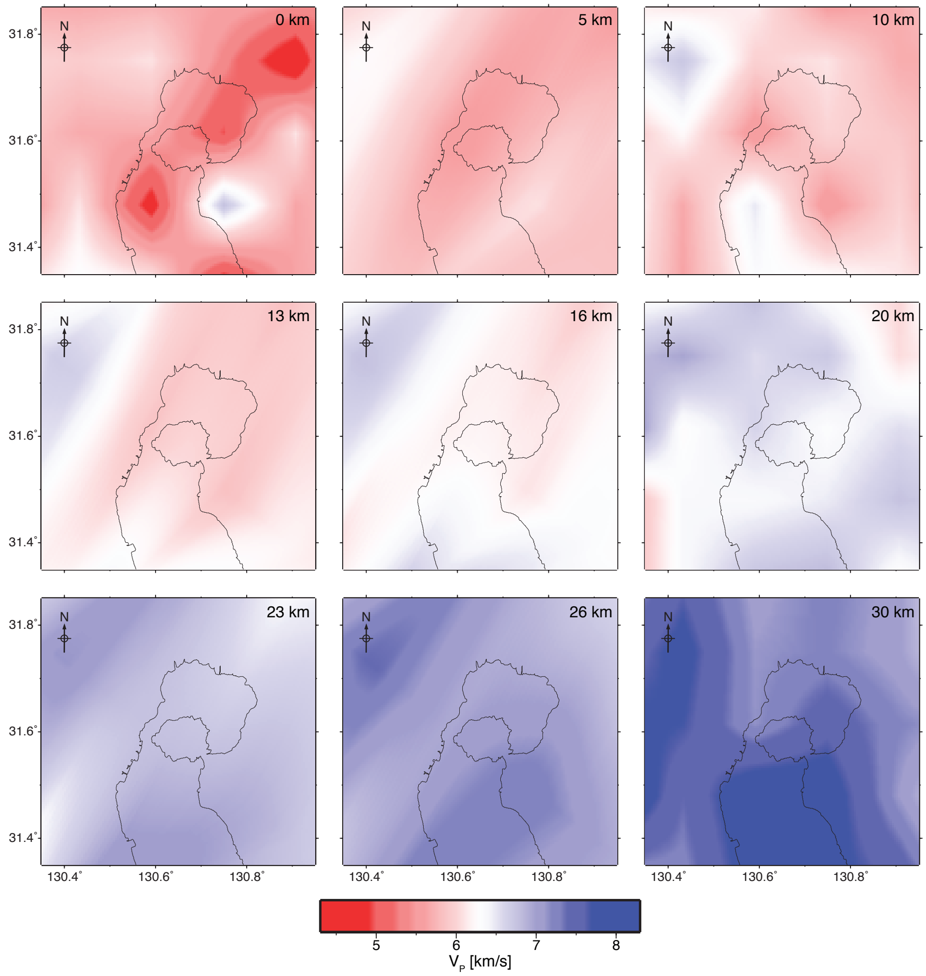

Supplementary Figure 2. **3D seismic tomography.** Colours show the distribution of seismic P-wave velocity. These data are used to constrain the Young's Modulus of the crust in the 3D model setup for a more realistic mechanical crustal representation, using equations (1) – (3). Maps created using GMT 4 [59].

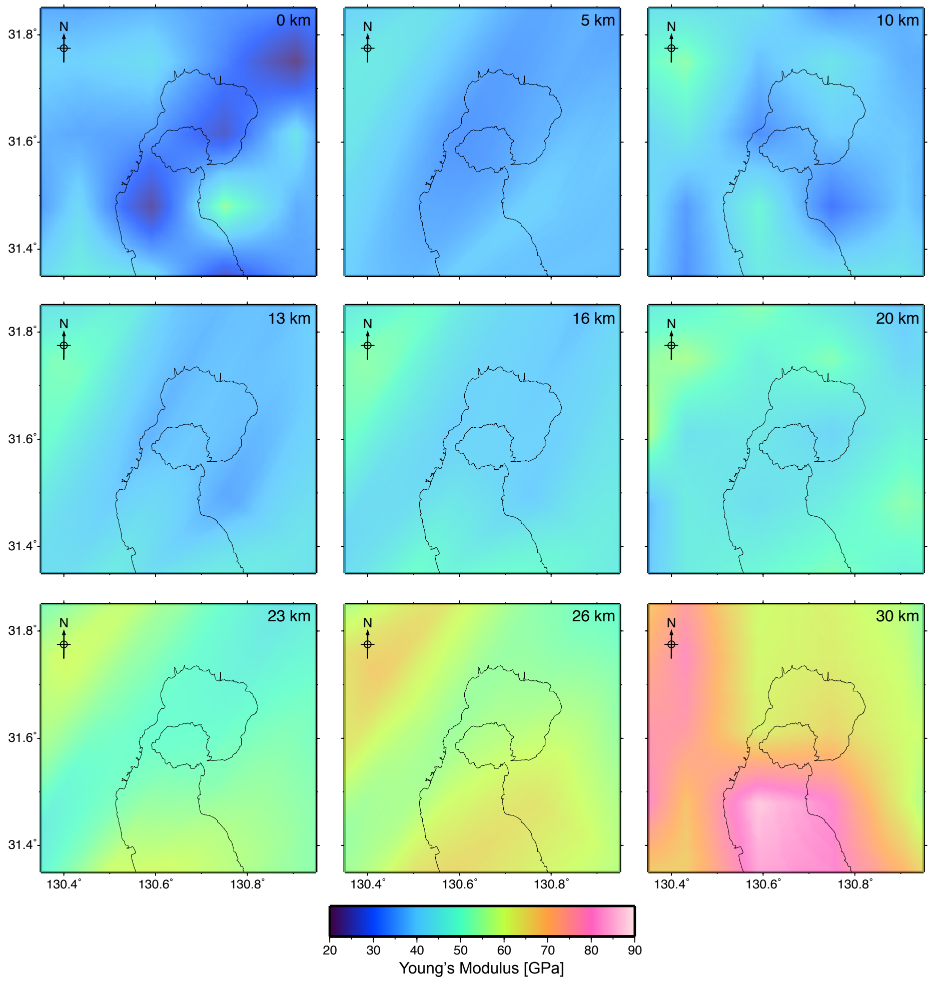

Supplementary Figure 3. **3D Young's Modulus distribution.** The 3D seismic tomography data (Fig. S2) are used to calculate the Young's Moduli (*Methods*, main text). There is an increase in Young's Modulus with depth and a region of lower Young's Modulus in the NE of Aira caldera. Maps created using GMT 4[59].

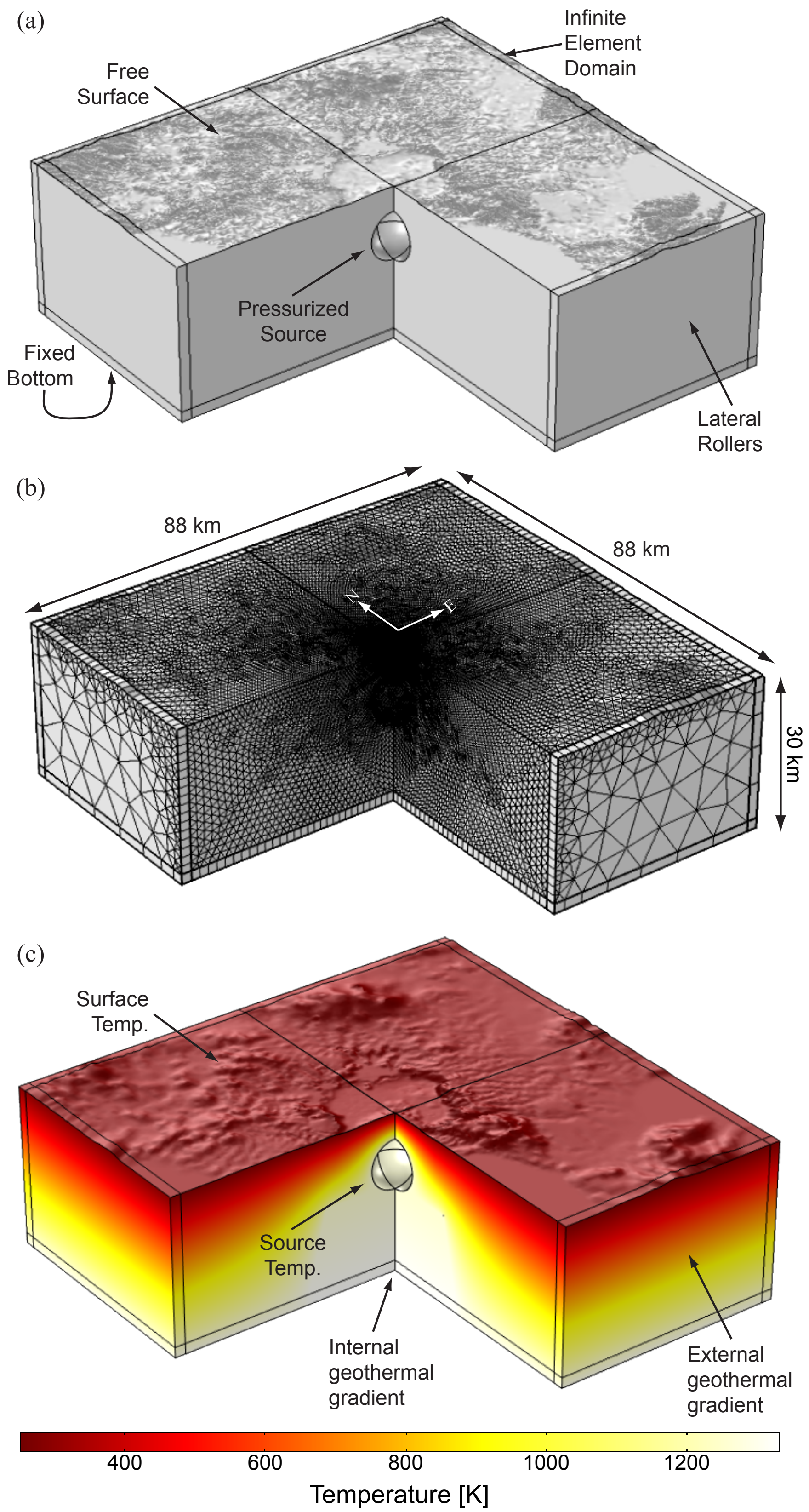

Supplementary Figure 4. The Finite Element model setup. (a) The model geometry and solid mechanics boundary conditions. See accompanying text for more details. (b) The Finite Element mesh, with higher mesh density around and above the source. (c) Thermomechanical model setup and heat transfer boundary conditions. The background geothermal gradient is perturbed by a hot (magmatic) source. A resulting temperature distribution is shown.

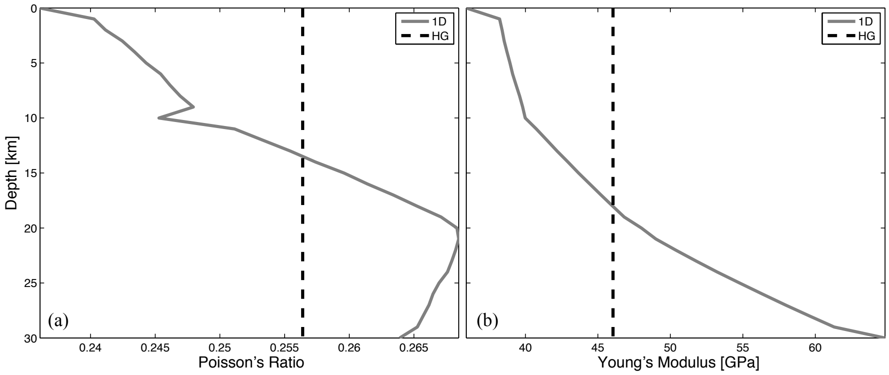

Supplementary Figure 5. Material property depth profiles. The variation in Poisson's Ratio (a) and Young's Modulus (b) with depth, in the 1D (grey solid line) and HG (black dashed line) model classes.

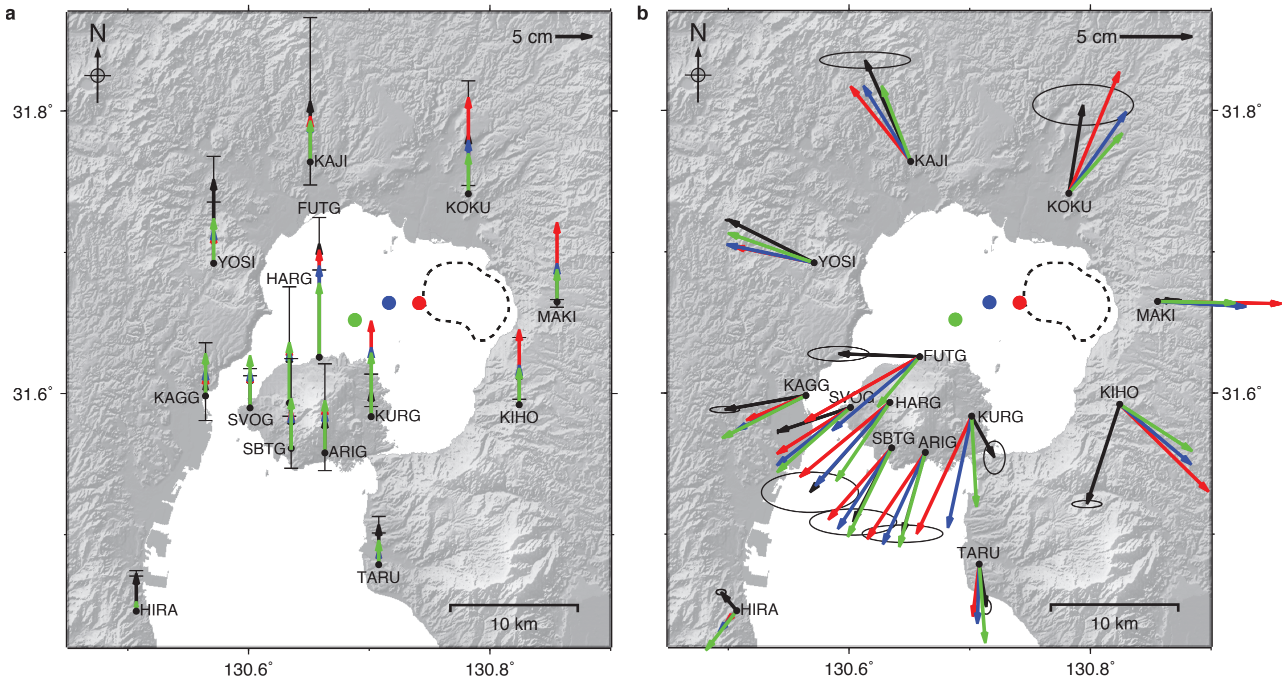

Supplementary Figure 6. Source shape comparison of 3D TOPO models. Black arrows indicate GPS measurements (1996 – 2007) with 95% confidence limits. The coloured arrows are the model predictions for the 3D TOPO model class with an oblate (red, same as Figure 3), spherical (blue), or prolate (green) shaped source, and a source volume equivalent to the final best-fit model (Methods, and Table 1), for vertical (**a**) and horizontal (**b**) components. The coloured circles indicate the horizontal source locations for each of the sources (same colour code). The oblate source has the smallest misfit value and best fit to the data. GPS station names are shown and the dashed line represents the outline of the Wakamiko depression. Maps created using GMT 4[59].

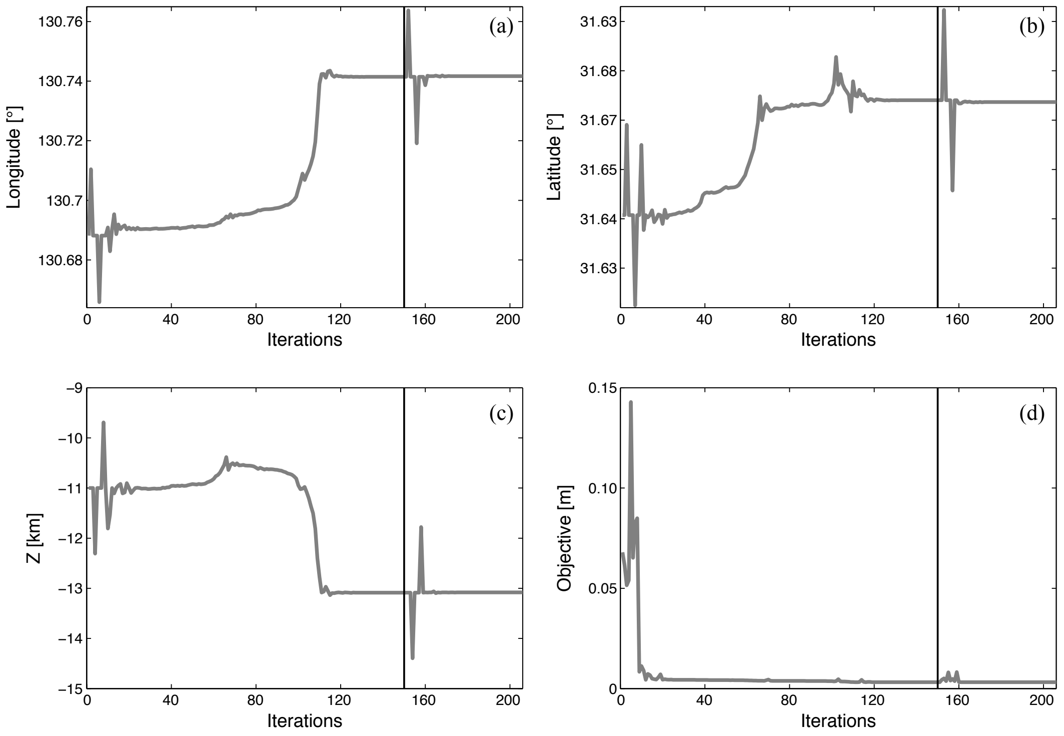

Supplementary Figure 7. Best-fit model convergence from subsequent inversions with nested parameter constraint grids. The first inversion varies the model parameters (X (a), Y (b), Z (c), and ΔP) within a wide parameter range to reduce the misfit objective function (d) (from an initial condition obtained via analytical models[17]). A subsequent inversion has a reduced parameter range, focused in on the results from the first inversion. It again varies the parameters to search for an improved solution, or ensure that the previous solution was optimal and robust. The latter is confirmed by the lack of further reduction in the misfit value (d). The vertical black line in the plots indicates the break between the first and second inversions.

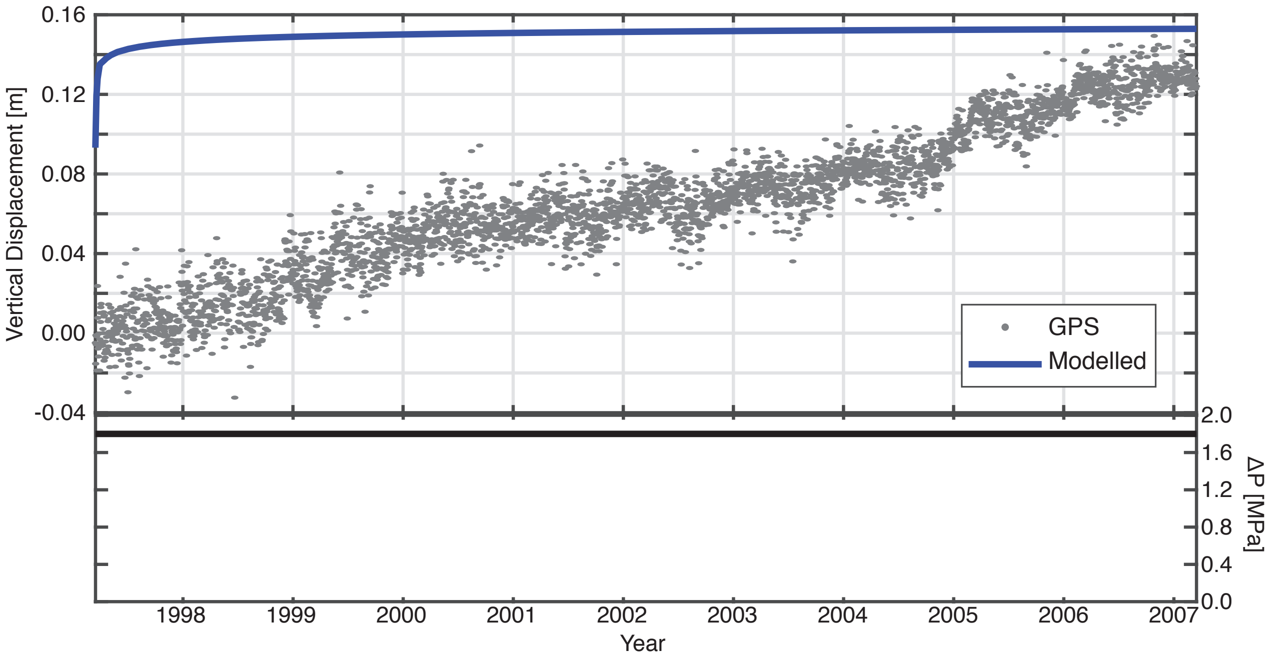

Supplementary Figure 8. Thermomechanical modelling with a constant pressure-time function. (Top) Grey dots indicate the observed vertical GPS displacement at the GEONET station, and the blue line shows the modelled deformation predictions using the TDVE model setup with a constant pressure-time function (in comparison to the ramped pressure time function in Fig 4 of the main text). The model predictions do not match the temporal inflation pattern, but do highlight the impact of viscoelastic deformation with a significant delayed displacement after the initial (instantaneous) elastic inflation. (Bottom) The constant pressure-time function applied.

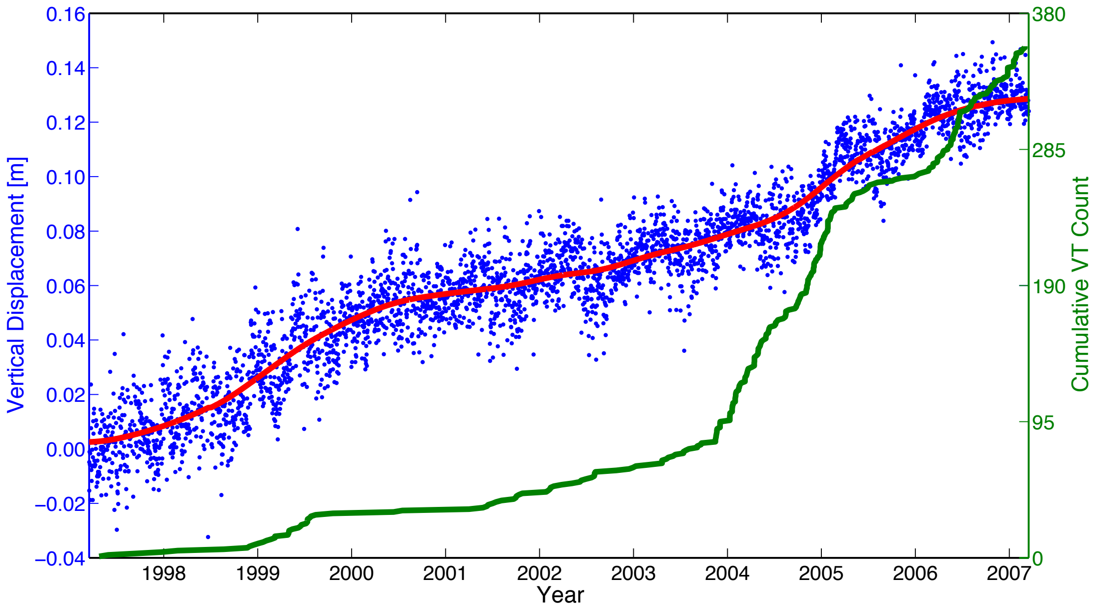

Supplementary Figure 9. The correlation between seismicity and deformation. Blue dots indicate the observed vertical GPS displacement at the GEONET station, and the red line shows the smoothed fit through this data. A correlation coefficient of ~0.95 indicates a strong relationship with the local volcano-seismicity rates (green line).

Supplementary Table 1. List of model parameters*.

Parameter	Definition	Value	Unit
V_P	P-Wave Velocity	4.37 – 8.23	km/s
V_S	S-Wave Velocity	2.57 – 4.65	km/s
ν	Poisson's Ratio	Eq. 1	-
ρ	Density	Eq. 2	g/cm^3
E_D	Dynamic Young's Modulus	Eq. 3	GPa
E	Young's Modulus	$E_D/2$	GPa
X	Longitude	m.v.	$^\circ$
Y	Latitude	m.v.	$^\circ$
Z	Depth to centre	m.v.	km
ΔP	Source Pressure	m.v.	MPa
ΔV	Source Volume Change	m.v.	m^3
R	Source Radius	m.v.	km
a	Vertical Axis	m.v.	km
b	Horizontal Axes	m.v.	km
V	Source Volume	m.v.	km^3
D_i	GPS Data	Fig. 3	m
E_i	GPS Error	Fig. 3	m
W_i	GPS Weight	Eq. 6	-
M_i	Modeled GPS	m.v.	m
J	Objective Function	Eq. 4	m
η	Viscosity	Eq. 8	Pa s
A_d	Dorn Parameter	10^9	Pa s
H	Activation Energy	135	kJ/mol
R	Universal Gas Constant	8.314	J/(mol K)
k	Thermal Conductivity	3	W/(m K)
C_p	Heat Capacity	1000	J/(kg K)
dT/dZ	Geothermal Gradients	34 & 70	K/km
T_{surf}	Surface Temp.	283	K
T_{mag}	Source Temp.	1273	K

*m.v. = Model variant

Supplementary Table 2. Tested source size parameters.

	R [km]	a [km]	b [km]	V [km^3]
Spherical	1	-	-	4.19
	2	-	-	33.51
	3	-	-	113.10
	4	-	-	268.08
	5	-	-	523.60
Prolate	-	2.08	0.69	4.19
	-	4.17	1.39	33.51
	-	6.24	2.08	113.10
	-	8.31	2.72	268.08
	-	10.41	3.47	523.60
Oblate	-	0.48	1.44	4.19
	-	0.96	2.88	33.51
	-	1.44	4.32	113.10
	-	1.92	5.76	268.08
	-	2.40	7.20	523.60

Supplementary Table 3. Inversion model t-test results (p-values). Bold values indicate statistical significance.

T-Test	*X*	*Y*	*Z*	*J*
Spherical				
TOPO: 3D vs 1D	0.1555	**0.0607**	0.7640	**0.0863**
TOPO: 3D vs HG	0.5559	**0.0208**	0.7199	**0.0373**
TOPO: 1D vs HG	0.2393	0.9289	0.5638	0.7180
HALF: 3D vs 1D	0.6321	0.1193	0.7627	**0.0062**
HALF: 3D vs HG	0.4728	**0.0090**	0.3453	**0.0184**
HALF: 1D vs HG	0.9165	0.7658	**0.0476**	**0.0434**
3D: TOPO vs HALF	**0.0314**	0.6286	0.7546	**0.0015**
1D: TOPO vs HALF	**0.0025**	0.5905	0.3472	**0.0001**
HG: TOPO vs HALF	**0.0022**	0.1137	0.3645	**$2.5\text{x}10^{-8}$**
Prolate				
TOPO: 3D vs 1D	0.7592	**0.0218**	0.7105	0.2239
TOPO: 3D vs HG	**0.0052**	**0.0005**	0.3584	0.4211
TOPO: 1D vs HG	0.1768	0.1843	0.7109	0.3576
HALF: 3D vs 1D	**0.0765**	**0.0001**	0.4135	**0.0044**
HALF: 3D vs HG	0.1159	**0.0002**	**0.0797**	**0.0054**
HALF: 1D vs HG	0.2746	0.6763	0.3745	0.4779
3D: TOPO vs HALF	**0.0195**	**$6.7\text{x}10^{-7}$**	0.3411	**0.0259**
1D: TOPO vs HALF	0.4906	**0.0002**	0.9384	0.1675
HG: TOPO vs HALF	**$1.9\text{x}10^{-5}$**	**$7.2\text{x}10^{-6}$**	0.5238	**0.0505**
Oblate				
TOPO: 3D vs 1D	0.4580	0.7543	0.5791	0.1580
TOPO: 3D vs HG	**0.0141**	**0.0341**	0.8093	**0.0245**
TOPO: 1D vs HG	**0.0343**	**0.0365**	0.6223	**0.0476**
HALF: 3D vs 1D	0.6126	**0.0621**	0.8446	0.2154
HALF: 3D vs HG	0.3618	**0.0770**	0.8914	0.1848
HALF: 1D vs HG	0.9281	0.3629	0.7536	0.6863
3D: TOPO vs HALF	0.6687	0.2151	0.6349	0.3863
1D: TOPO vs HALF	0.8744	0.1224	0.1951	0.6266
HG: TOPO vs HALF	**0.0531**	**0.0806**	0.5063	**0.0643**
